# Supplementary material for: SARS-CoV-2 Papain-like Protease Negatively Regulates the NLRP3 Inflammasome Pathway and Pyroptosis by Reducing the Oligomerization and Ubiquitination of ASC
Source: Microorganisms. 2023 Nov 17;11(11):2799. doi: 10.3390/microorganisms11112799 (PMC10673202; doi:10.3390/microorganisms11112799)
Supplement: Supplementary file 1 [file microorganisms-11-02799-s001.zip › SourceData of supplemental files.pdf]

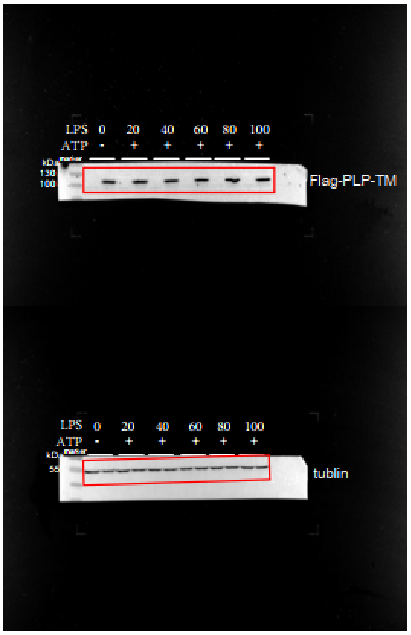

SourceData Figure S2

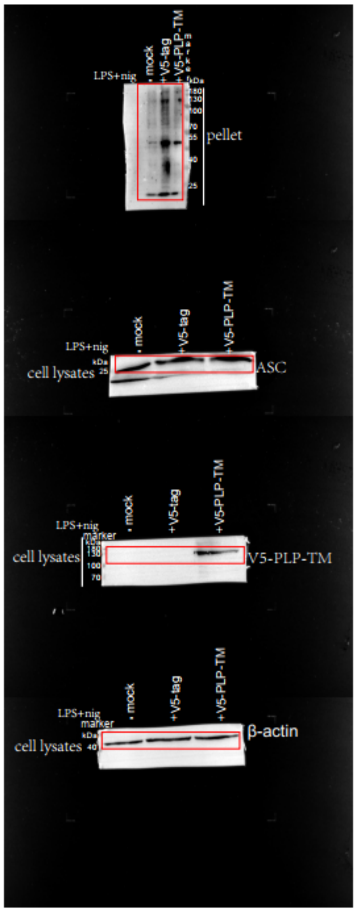

SourceData Figure S4

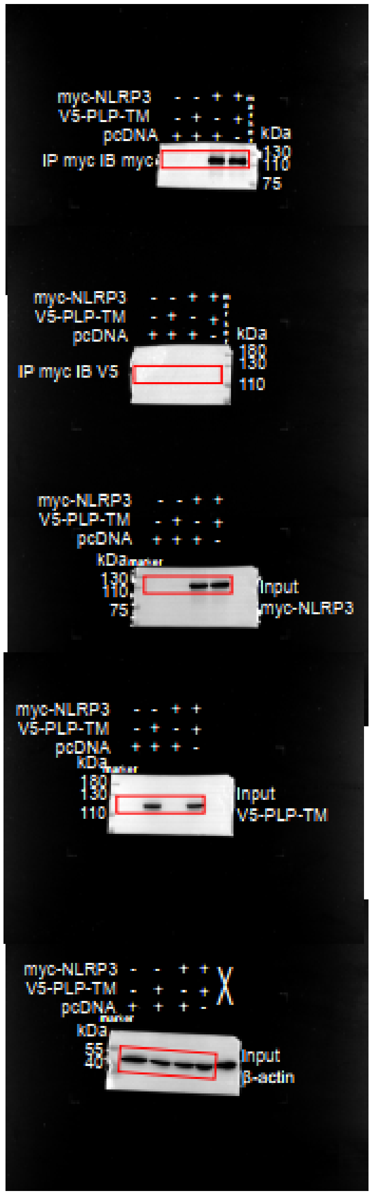

SourceData Figure S5A

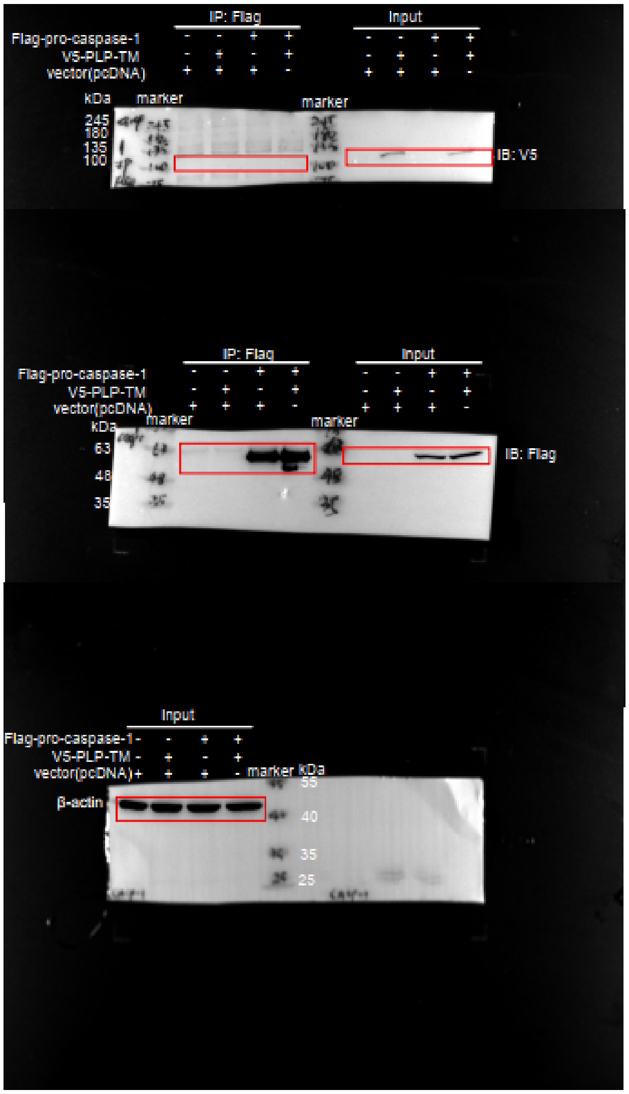

SourceDataF5B

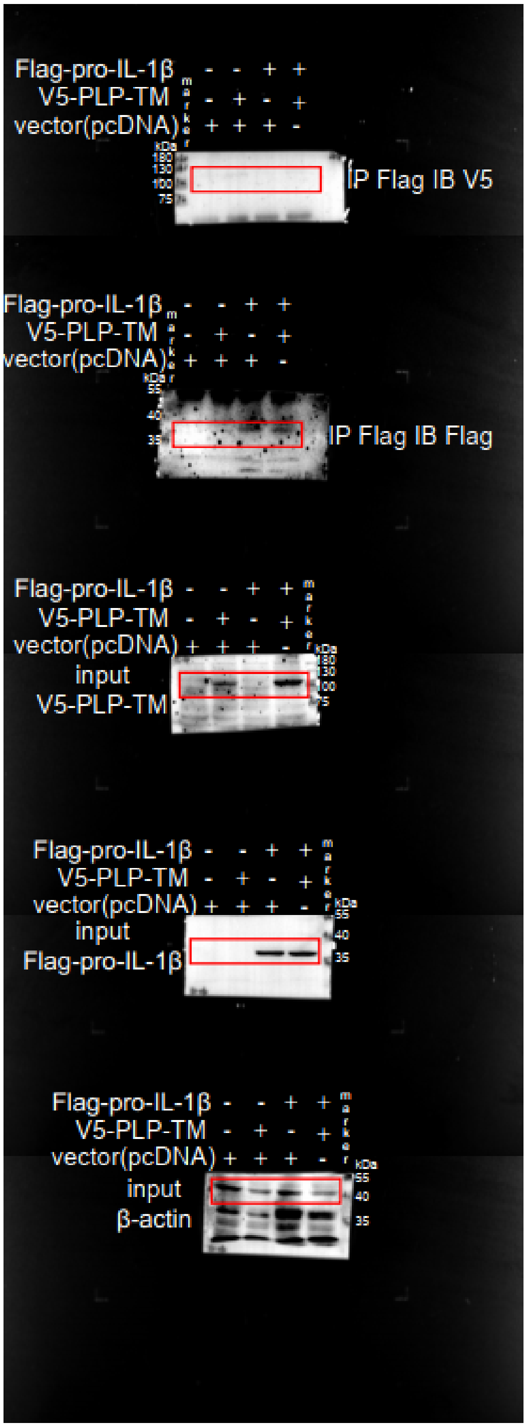

SourceData Figure S5C
