# Supplementary material for: SARS-CoV-2 Papain-like Protease Negatively Regulates the NLRP3 Inflammasome Pathway and Pyroptosis by Reducing the Oligomerization and Ubiquitination of ASC
Source: Microorganisms. 2023 Nov 17;11(11):2799. doi: 10.3390/microorganisms11112799 (PMC10673202; doi:10.3390/microorganisms11112799)
Supplement: Supplementary file 1 [file microorganisms-11-02799-s001.zip › Supplementary Materials.pdf]

Supplementary Materials:

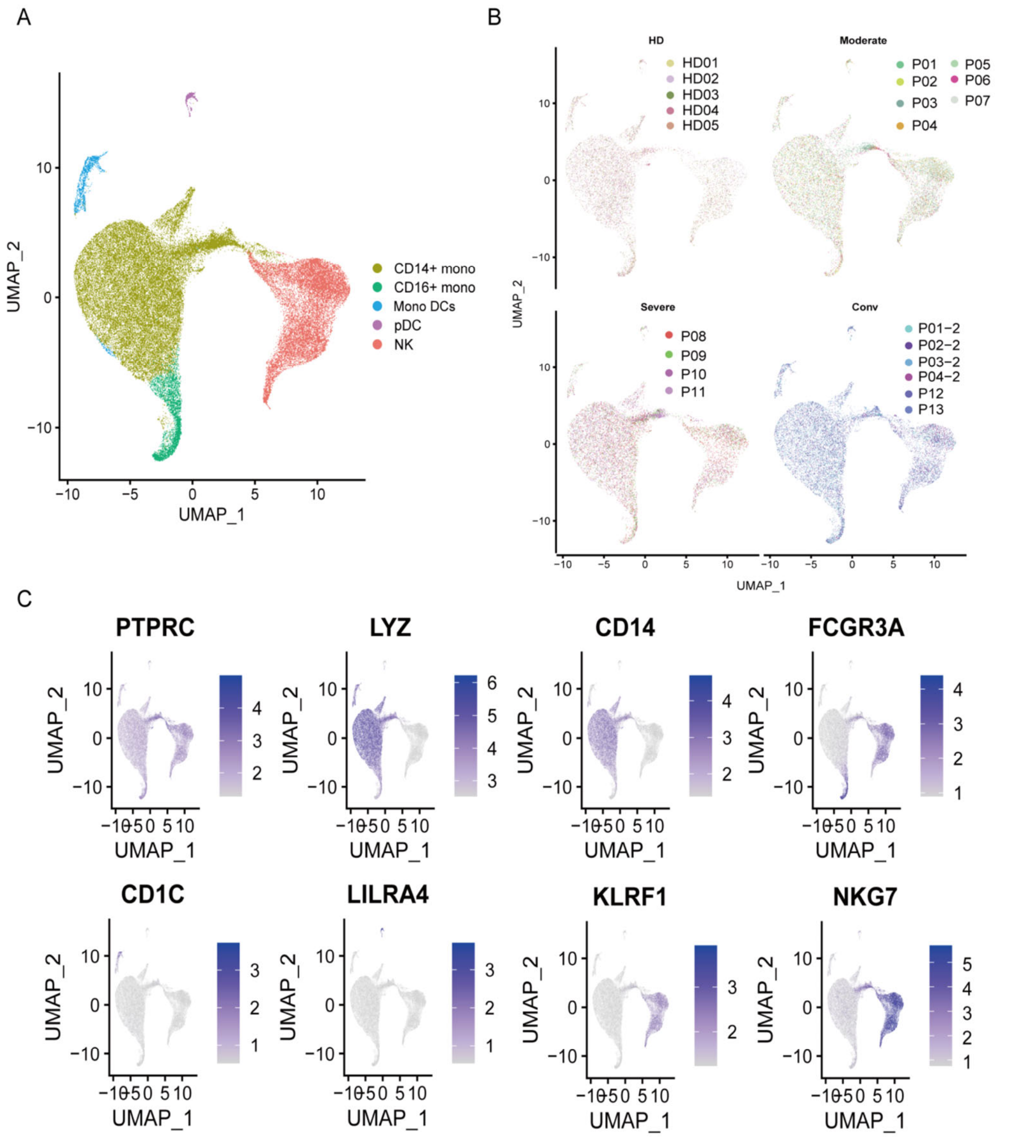

Figure S1. Identification and annotation of monocytes, dendritic cells, and natural killer (NK) cells from Peripheral blood mononuclear cells (PBMCs) of the COVID-19 patients and healthy controls. (A) UMAP representation of identified monocytes, dendritic cells, and NK cells, with every cell colored according to its cell type. (B) UMAP plots show the information of cells' sample origin. Every cell was depicted in a specific color to indicate its original sample information. (C) UMAP projection of the expression levels of canonical markers of $CD14^+$ monocytes (LYZ^+CD14^+), CD16+monocytes ($LYZ^+FCGR3A^+$), monocyte-derived dendritic cells ($CD1C^+$), plasmacytoid dendritic cells ($LILRA4^+$), and NK cells ($KLRF1^+$).

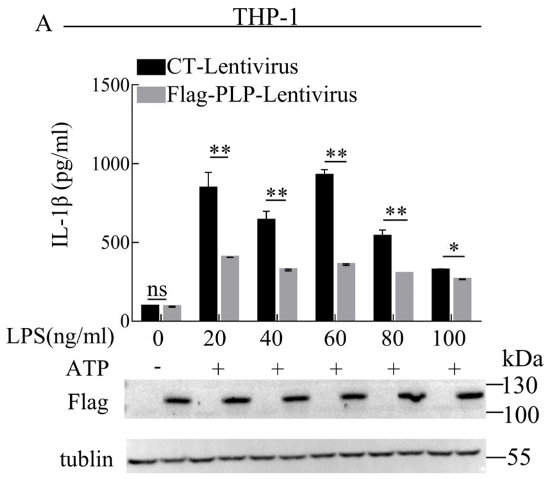

Figure S2. SARS-CoV-2 PLP reduces the expression of IL-1β (p17) in HEK-293T and THP-1 cells. (A) SARS-CoV-2 PLP reduces the secretion of IL-1β with different concentrations of LPS stimulation.

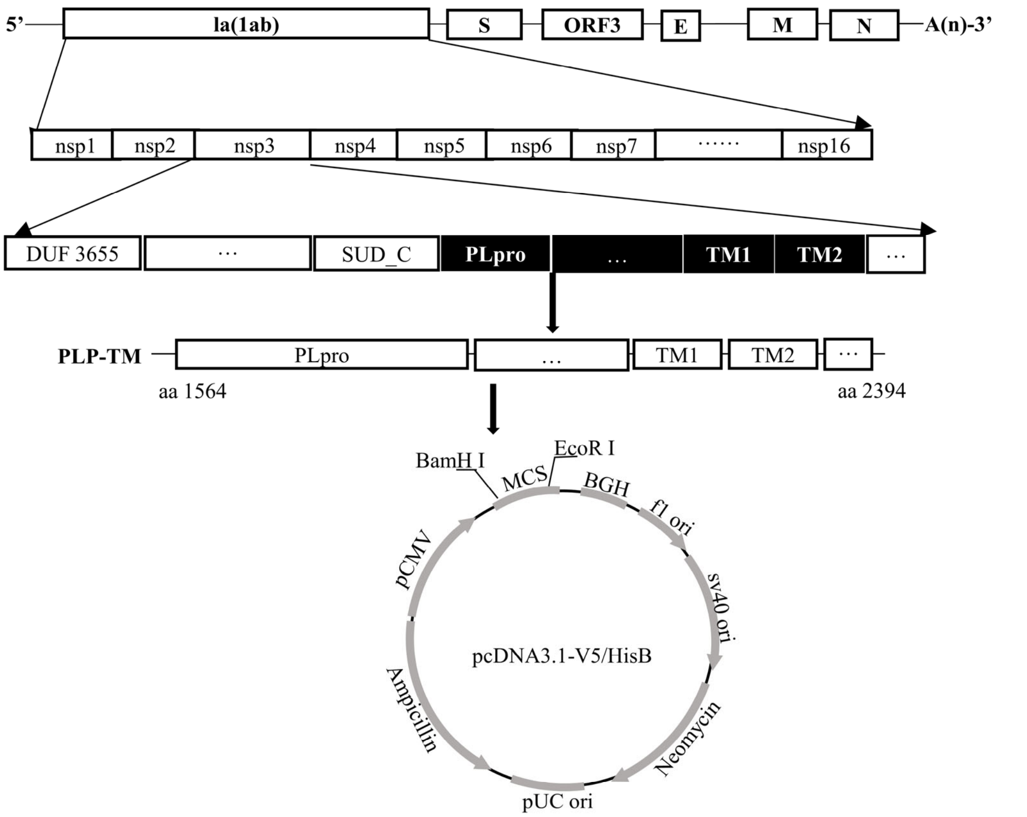

Figure S3. The construction of SARS-CoV-2 PLP-TM expressing component. The PLP-TM encoding nucleotide sequence (aa1564-2394 in pp1ab) of SARS-CoV-2 Wuhan-Hu-1 (GenBank accession number NC_045512.2) was synthesized after codon optimization and cloned into pcDNA3.1-V5/HisB plasmid between BamHI and EcoRI.

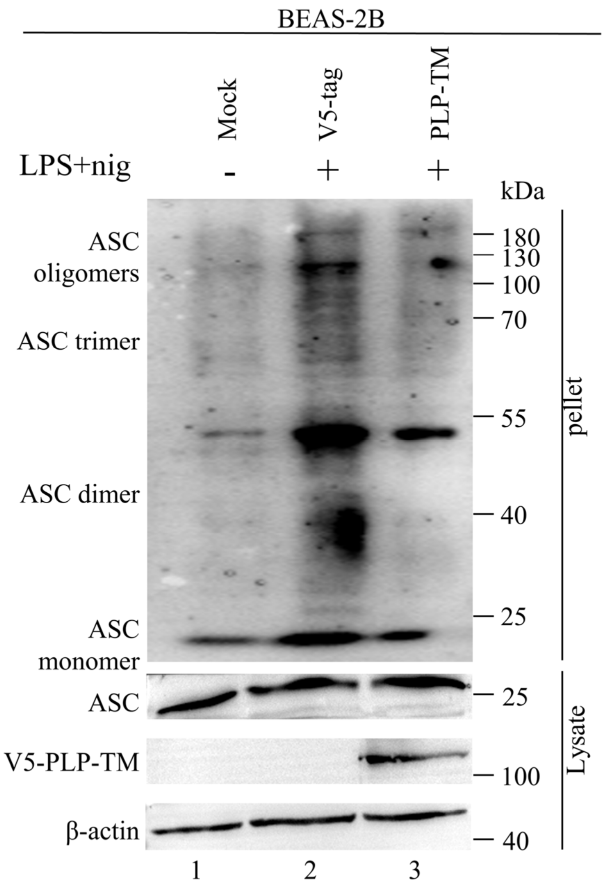

Figure S4 SARS-CoV-2 PLP interrupts the oligomerization of ASC in BEAS-2B cells. BEAS-2B cells were transfected with V5-vector or V5-SARS-CoV-2 PLP-TM for 24h and stimulated with 1ug/ml LPS for 12h plus 10μmol/L nigericin for 1.5h. Cell lysates were collected for the detection of ASC oligomerization.

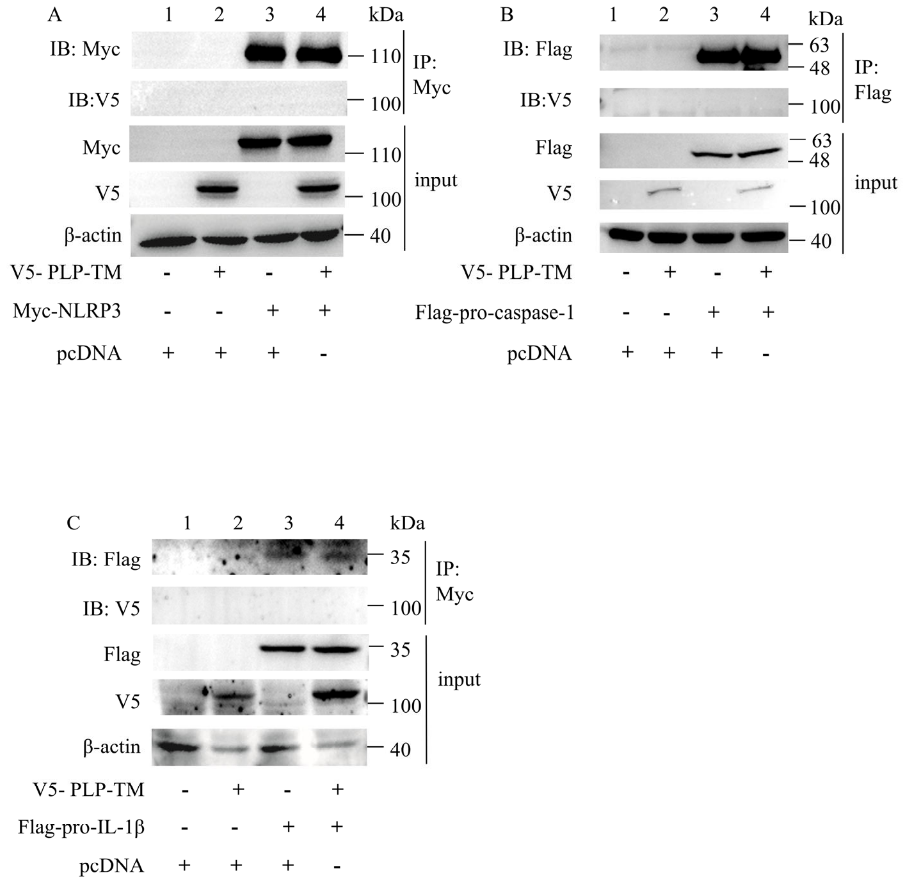

Figure S5 SARS CoV-2 PLP-TM did not interact with NLRP3/caspase-1/IL-1β. (A) HEK-293T cells were transfected with plasmids encoding Myc-tagged NLRP3 (lanes 3 and 4), or with plasmids encoding V5-tagged SARS CoV-2 PLP-TM (lanes 2 and 4) and with an empty V5-tagged vector. Cell lysates were collected and immunoprecipitated with antibodies against Myc, followed by immunodetection with antibodies against V5. Immunoblot analysis of cell lysates was conducted as indicated. (B) HEK-293T cells were transfected with plasmids encoding Myc-tagged pro-caspase-1 (lanes 3 and 4), or with plasmids encoding V5-tagged SARS CoV-2 PLP-TM (lanes 2 and 4) and with an empty V5-tagged vector. Cell lysates were collected and immunoprecipitated with antibodies against Myc, followed by immunodetection with antibodies against V5. Immunoblot analysis of cell lysates was conducted as indicated. (C) HEK-293T cells were transfected with plasmids encoding Flag-tagged pro-IL-1β (lanes 3 and 4), or with plasmids encoding V5-tagged SARS CoV-2 PLP-TM (lanes 2 and 4) and with an empty V5-tagged vector. Cell lysates were collected and immunoprecipitated with antibodies against Myc, followed by immunodetection with antibodies against V5. Immunoblot analysis of cell lysates was conducted as indicated.
